# Implicit Self-Esteem Decreases in Adolescence: A Cross-Sectional Study

**DOI:** 10.1371/journal.pone.0089988

**Published:** 2014-02-25

**Authors:** Huajian Cai, Mingzheng Wu, Yu L. L. Luo, Jing Yang

**Affiliations:** 1 Key Laboratory of Behavioral Science, Institute of Psychology, Chinese Academy of Sciences, Beijing, China; 2 Department of Psychology, Zhejiang University, Hangzhou, China; University of Bologna, Italy

## Abstract

Implicit self-esteem has remained an active research topic in both the areas of implicit social cognition and self-esteem in recent decades. The purpose of this study is to explore the development of implicit self-esteem in adolescents. A total of 599 adolescents from junior and senior high schools in East China participated in the study. They ranged in age from 11 to 18 years with a mean age of 14.10 (*SD* = 2.16). The degree of implicit self-esteem was assessed using the Implicit Association Test (IAT) with the improved *D* score as the index. Participants also completed the Rosenberg Self-Esteem Scale (α = 0.77). For all surveyed ages, implicit self-esteem was positively biased, all *t*s>8.59, all *p*s<0.001. The simple correlation between implicit self-esteem and age was significant, *r* = −.25, *p* = 1.0×10^−10^. A regression with implicit self-esteem as the criterion variable, and age, gender, and age × gender interaction as predictors further revealed the significant negative linear relationship between age and implicit self-esteem, *β* = −0.19, *t* = −3.20, *p* = 0.001. However, explicit self-esteem manifested a reverse “U” shape throughout adolescence. Implicit self-esteem in adolescence manifests a declining trend with increasing age, suggesting that it is sensitive to developmental or age-related changes. This finding enriches our understanding of the development of implicit social cognition.

## Introduction

Since the first theoretical documentation by Greenwald & Banaji [1], implicit self-esteem has remained an active research topic in both the areas of implicit social cognition and self-esteem. Research has established that implicit self-esteem is, at most, weakly associated with explicit self-esteem [2], [3] and positive in nature regardless of culture, ethnicity, and age [4], [5]. Also, research has linked low implicit self-esteem to negative experiences and demonstrated its moderating role in various relationships, such as reactions to threat [6], [7]. Given the importance of implicit self-esteem and burgeoning interest in the development of implicit social cognition [4], [8], [9], it is imperative to understand how implicit self-esteem develops and whether it differs as a function of age or developmental period. Research from a developmental perspective may help us understand not only the developmental trajectory of implicit self-esteem, but also the relationship between explicit and implicit self-esteem as well as how to maintain optimal implicit self-esteem. Currently most studies have focused on implicit self-esteem among adults. In contrast, research on the development of implicit self-esteem is still limited. The purpose of the current study was to explore the development of implicit self-esteem among adolescents.

As an important form of implicit social cognition, “the introspectively unidentified (or inaccurately identified) trace of past experience” [1], implicit self-esteem has been widely accepted as a type of automatic and over-learned self-evaluation [2], [10]. Theories such as experiential system theory [11] and attachment theory [12] suggest that implicit self-esteem is primitive and formed early in one’s life. Consistent with these perspectives, research has shown that implicit self-esteem is associated with early life experiences [13] and children in primary school exhibit positive implicit self-esteem [4].

To our knowledge, only a few studies have examined the effect of age difference on implicit self-esteem [4], [14], [15]. In one study, Hoorens et al. [15] investigated implicit self-esteem by examining preferences for name letters among primary school students in grades 2, 4, and 6 and found an increasing trend of implicit self-esteem in the primary school grades. In another study, Hummert et al. [14] examined differences in implicit self-esteem based on age by using the Implicit Association Test (IAT) [3] among three age groups: young adults (18–29 years), young-old adults (55–74 years) and old-old adults (75–93 years) and found comparable levels of positive implicit self-esteem across these three age groups. More recently, Dunham et al. [4] simultaneously investigated three types of implicit social cognition (self-esteem, group identity, and group attitude) in Hispanic American children (aged 5 to 12) and adults. The Implicit Association Test (IAT) adapted for use with children was employed as a measure of implicit social cognition. Results revealed pronounced implicit self-positivity in both children and adults, although the self-positivity was still relatively low compared with other majority samples [3]. More notably, the level of this implicit bias was comparable among children of different ages and appeared to be similar between children and adults. In summary, findings from these few studies provided some preliminary knowledge about the development of implicit self-esteem throughout the span of a person’s life. However, research examining the impact of age difference on implicit self-esteem in adolescents is still lacking.

Generally speaking, adolescence, depicted as a period of “storm and stress” [16], [17], represents a dramatic developmental transition from childhood to adulthood. Increasing negative affect and conflict with parents typically characterize individuals going through this phase [16], [18]–[21]. Affective experiences and interactions with significant others constitute important sources of implicit attitude [10], [13], [22]. Research has shown that negative affect and conflict with parents are deleterious for implicit self-esteem [13], [23], [24]. Given this circumstance, we may expect that after entering into adolescence, young people would experience a decline in implicit self-esteem due to increasing negative affect as well as conflict with parents.

More specifically, according to self-development theory [25], the acquisition of a formal thinking ability from childhood to adolescence is a critical developmental change. The increasing formal thinking ability can greatly enhance adolescents’ capacity to evaluate the self as an abstract and distinct being from a third person perspective and to incorporate others’ opinions as well as social comparison information into their own self-judgment. Collectively, these developments aid in forming an increasingly realistic self-view [25], [26]. In consideration of the fact that children typically have a highly inflated self-view [26], we may also infer that implicit self-esteem, as kind of over-learned self-evaluation, would decline during adolescence.

In all, we expected that implicit self-esteem would decrease with increasing age during adolescence. To test this, we conducted a cross-sectional study among adolescents aged 11 to 18 years old. We used IAT to measure implicit self-esteem [3], [27]. The IAT has desirable psychometric properties compared with other implicit measures [2] and has been the most widely used measure of implicit self-esteem [6]. Also, most studies involving the development of implicit social cognition have used the IAT [4], [9]. For the purposes of comparison, we included an explicit self-esteem measure. Explicit self-esteem could be jointly influenced by the developmental process and by external social factors, such as social desirability and social comparison [28], [29]. Indeed, findings on the development of explicit self-esteem during adolescence are inconclusive [30]. As a result, we have not included any specific predictions about the development of explicit self-esteem here. In examining the relationship between age and self-esteem, we controlled for gender and its interaction with age because self-esteem for male and females may not follow the same developmental trajectory [25].

## Methods

### Ethics Statement

Ethical approval for the study has been provided by the Ethics Committee of the Institute of Psychology, Chinese Academy of Sciences. Prior to the study, we obtained written informed consent from the adolescents and their parents.

### Participants

A total of 599 adolescents from two middle schools (comprised of both junior and senior high school aged students) in East China participated in the study. They ranged in age from 11 to 18 years with a mean age of 14.10 (*SD* = 2.16). Gender composition of the sample is presented in Table 1.

**Table 1:** The Means (SDs) of and correlations between explicit and implicit self-esteem.

Age	N(male)	Mean (*SD*)		Correlation
		Explicit	Implicit	
11	54(27)	2.87(0.43)	0.58(0.37)	0.03
12	123(61)	2.98(0.38)	0.58(0.39)	−0.07
13	109(59)	3.06(0.48)	0.51(0.39)	−0.08
14	90(54)	3.07(0.48)	0.49(0.42)	0.04
15	42(26)	2.30(0.53)	0.55(0.42)	0.23
16	55(25)	2.88(0.32)	0.36(0.26)	−0.26
17	80(28)	2.90(0.40)	0.33(0.28)	0.07
18	46(22)	2.78(0.45)	0.32(0.24)	0.07
Total	599(297)	2.96(0.44)	0.48 (0.37)	0.03

### Measures and Procedure

All participants completed the explicit self-esteem and implicit self-esteem measures on computers in individual, quiet rooms. The order of administering explicit and implicit measures was counterbalanced. We assessed explicit self-esteem with the Rosenberg Self-Esteem Scale [31]. This scale has been well validated and widely used in China [32]. It includes 10 statements such as “I take a positive attitude toward myself” and “At times I think I am no good at all”. Participants indicated the extent to which they agree or disagree with each statement on a 4-point Likert scale (1 = *strongly disagree*, 4 = *strongly agree*). In this sample, the internal consistency was desirable (α = 0.77).

We assessed implicit self-esteem using IAT [3]. Just like the standard IAT [27], [33], we used 7 blocks in the self-esteem IAT. There are two types of critical blocks: in the *self+pleasant* blocks, participants were instructed to map self-related (mine, my, me, I) and pleasant (beautiful, lovely, valuable, attractive, smart) stimuli onto one computer key, and other-related (his, he, they, their) and unpleasant stimuli (ugly, useless, stupid, banal, disgusting) to another key (20 practice trials, 40 test trials); in the *self+unpleasant* blocks, participants were asked to map self-related and unpleasant stimuli onto one computer key, and other-related and pleasant stimuli to another key (20 practice trials, 40 test trials). Following the standard procedure of IAT [27] in administering the test, the two critical blocks were counterbalanced across participants. Thus, half of the participants completed the *self+pleasant* block first and then the *self+unpleasant* block, while the other half completed the two blocks in reverse order. In all blocks, participants were asked to respond rapidly and with few errors. The performance difference between the two types of critical blocks, each of which included 60 trials in total, was taken as the index of implicit self-esteem: positive implicit self-esteem was indicated by faster performance in the *self+pleasant* blocks. Modeling previous studies [34], we used the improved *D* score as the index of implicit self-esteem. To compute the *D* score, we used latencies from all four critical blocks. The trials with latency greater than 10000 ms were discarded. The mean latencies for the *self+pleasant* and *self+unpleasant* blocks were computed based on all remaining trials, respectively. The pooled standard deviation was computed using latency from all trials with a correct response. Finally, the *D* was obtained by dividing the mean latency difference between the two types of critical blocks with pooled standard deviation [33], [34]. The split-half reliability was 0.63, which is comparable to findings in previous studies [33], [34].

## Results

**Figure 1:**
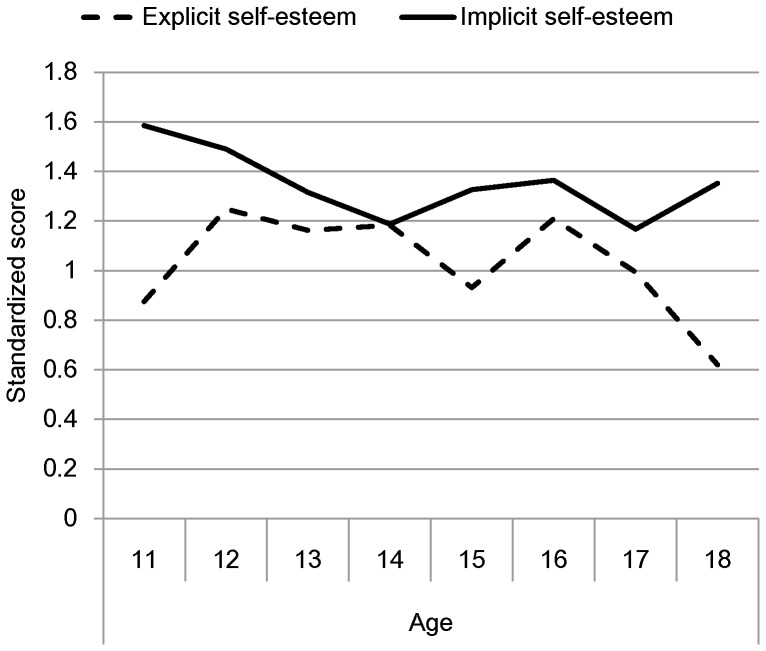
The standardized implicit and explicit self-esteem by ages.

We displayed the descriptive statistics for implicit self-esteem and explicit self-esteem across all ages in Table 1. To facilitate simultaneous visualization of the developmental trends of implicit and explicit self-esteem, the mean raw scores for each age were standardized with the scale midpoints, 0 and 2.5, as references, and overall *SD*s, 0.24 and 0.44, as units, respectively. Then the standardized scores were plotted in Figure 1.

### Implicit Self-esteem

All IAT means were significantly greater than zero, all *t*s>8.59, all *p*s<0.001, suggesting adolescents of all ages have positive implicit self-esteem. Levels of implicit self-esteem, however, were lower among older rather than younger adolescents, as revealed by the simple correlation between implicit self-esteem and age, *r* = −.25, *p*<0.001 (also see Figure 1). Furthermore, we conducted a regression with implicit self-esteem as the criterion variable, and age, gender, and age × gender interaction as the predictors [35]. The results revealed a significant negative linear trend of age on implicit self-esteem, *β* = −0.19, *t* = −3.20, *p*<0.001. With increasing age, implicit self-esteem decreased from 0.58 by the age of 11 to 0.32 at 18 years, *d* = 0.85. Neither the gender effect (*β* = −0.05, *t* = −1.20, *p* = 0.23) nor the interaction (*β* = −0.08, *t* = −1.38, *p* = 0.17) was significant.

### Explicit Self-esteem

Consistent with previous findings [32], means of explicit self-esteem for all ages came in significantly above the scale midpoint (2.5), all *t*s>4.2, all *p*s<0.001. Although the simple correlation between explicit self-esteem and age was significant, *r* = −0.10, *p*<0.05, visual inspection suggested a possible quadratic trend (see Figure 1). To further explore the relation between age and explicit self-esteem, we conducted a regression analysis with explicit self-esteem as the criterion variable, and age, gender, age × gender interaction, and squared age as the predictors [35]. The effects of age, gender, and age × gender interaction were not significant: *β*s = −0.04, −0.04, 0.01, *ts* = −0.96, −0.57, 0.11, *ps* = 0.34, 0.57, 0.91, respectively. The quadratic effect of age, however, proved to be significant, *β* = −0.17, *t* = −3.86, *p*<0.001.

### The Relationship between Implicit and Explicit Self-esteem

Additionally, we examined the correlation between explicit and implicit self-esteem. As shown in Table 1, correlations were non-significant for each age group, individually as well as across the total sample, all *t*s<1.96, all *p*s>0.05, showing that explicit and implicit self-esteem were at most very weakly related in this adolescent sample. None of the correlations significantly differed from the others except for differences exhibited between the ages of 15 and 16, *z* = 2.37, *p*<0.05, which was somewhat unexpected.

## Discussion

Numerous studies have examined the development of self-esteem tapped by a self-report measure and have revealed a declining trend from childhood to adolescence [26], but an increasing trend from adolescence to adulthood [36]. Research on the development of implicit self-esteem, however, is still very limited. We examined age trends in implicit self-esteem during adolescence from the ages of 11 to 18. Consistent with previous findings, implicit self-esteem during adolescence was positive and largely dissociated from explicit self-esteem [2]. Interestingly, a declining trend for implicit self-esteem was found. In contrast to implicit self-esteem, an inverse ‘U’ shape trend was identified for explicit self-esteem, that is, rising first and declining later.

Our research is the first to reveal the developmental trend that relates to implicit self-esteem during adolescence. Some dual-process theories have suggested that implicit self-esteem may be formed early and that it resists developmental change [11], [37]. Some extant studies also suggest that implicit self-esteem is unchanged during childhood (5 to 9 years old) and even throughout the whole of adulthood [4], [14]. However, our findings revealed that implicit self-esteem decreases as age increases during adolescence. This declining trend is consistent with the self-development theory [25] and the “storm and stress” account [17]. Previous studies also have shown that implicit self-esteem is sensitive to momentary situational changes and long term acculturation as well as self-regulation. For example, Baccus et al. [38] found that pairing self-associated stimuli with positive stimuli could increase implicit self-esteem instantly. From a cultural perspective, Hetts et al. [39] found that exposure to an individualistic culture would lead to an increase in implicit self-esteem among people from a collectivistic culture. In another case, Jones et al. [40] found that a threat to one’s self-concept would lead to a decrease in implicit self-esteem among people with low self-esteem, whereas for people with high self-esteem, results registered an increase in implicit self-esteem. We have established further that implicit self-esteem also changes as a function of developmental or age-related shifts, extending our understanding of the malleability of implicit self-esteem.

These findings enrich our overall understanding of the development of self-esteem in adolescence. Although numerous studies have been conducted on the development of self-esteem in adolescence, all have focused on explicit self-esteem. Studies of implicit self-esteem, in contrast, are limited. For instance, Gregg and Sedikides [41] examined late adolescence at age 17. Verkuyten [42] explored early adolescence between ages 10 and 13. Neither of these studies, however, took age differences into account. In this study, therefore, we examined age differences in implicit self-esteem across the age span from 11 to 18 years old. We found that implicit self-esteem was largely independent of explicit self-esteem during adolescence. This outcome is consistent with previous studies that observed this relationship is weak in adult samples [2], [3] as well as with norms suggested by the dual-processes theories [11], [37]. Moreover, we have discovered that implicit self-esteem tends to follow a different trajectory. While explicit self-esteem increases first and then declines later, implicit self-esteem steadily declines. The differential development patterns may suggest that biological, psychological, and social transformations cause differential impact on the development of explicit and implicit self-esteem and consequently result in distinct developmental trajectories. Developmental shifts may also imply that some factors other than self-development, such as social comparison information and social desirability, have influenced the results obtained from explicit measure, particularly in the East where the self is highly social oriented [31], [43]. In all, our findings suggest that both explicit and implicit perspectives are important in order to fully understand developmental changes in self-esteem throughout adolescence.

Consistent with previous findings, apparent positivity of implicit self-esteem was observed across all ages during adolescence. Along with the findings of Dunham et al. [4], our study conveys that the previously established prevalence of implicit self-positivity in adults, regardless of culture and ethnicity [4], [5], [44], also proves to be true for younger ages. Regarding the lifespan development of implicit self-esteem, findings from our study combined with those of previous studies [4], [14], [15] suggest that although implicit self-esteem could be relatively stable across major development stages [4], [14], changes within specific developmental periods may still occur, such as an increase during childhood [15] but a decrease in adolescence. More studies, nevertheless, are needed to confirm these preliminary results.

Since the IAT is a reaction time-based categorization task, some may conclude that the basic cognitive and response speed would confound the IAT effect and contribute, at least partly, to the declining trend of implicit self-esteem we found. Indeed, we noted a negative correlation between age and overall response latency (*r* = −0.19, *p*<.001), suggesting that adolescent response is faster as age increases. Past research has determined that response speed is negatively associated with the reaction time-based IAT score [45] and may constitute a possible confound in age difference [14]. However, research also showed that the improved *D* measure is able to minimize the confounding effect of the response speed [14], [46]. Consious of this circumstance, we used the *D* measure as an index of implicit self-esteem. The possibility that the declining trend of the IAT effect is caused by response speed-related cognitive skills should be minimal.

A few limitations to our study are noteworthy. One limitation of the current study is its cross-sectional design, which makes it difficult to distinguish cohort from developmental effects as well as to identify the specific factors responsible for changes in explicit and implicit self-esteem. Research employing a longitudinal design is definitely required in the future. Second, we only examined implicit self-esteem tapped by IAT, leaving open the question of whether these findings will generalize to other measures. Research using diverse implicit measures is therefore also needed. Third, although the overall sample of the current study is large, the sample size is relatively small for each age group. Replication with larger samples is desirable in the future. Finally, given the study was conducted in China with the potential influence of culture, replications with samples from other cultures are also desirable [43]. Despite these limitations, the study is useful in providing the first delineation of developmental change in implicit self-esteem during adolescence, highlighting the importance of studying implicit self-esteem throughout an entire lifespan.
